# Folate-Functionalized Polymeric Nanoparticles for 5‑Fluorouracil Delivery to Prostate Cancer: Physicochemical and In Vitro/In Vivo Characterization

**DOI:** 10.1021/acsomega.5c07466

**Published:** 2025-12-25

**Authors:** Bhumi Bhatt, Gajanan Kalyankar, Bhavin Vyas, Manisha Lalan, Nimeet Desai, Lalitkumar K. Vora, Pranav Shah

**Affiliations:** † Maliba Pharmacy College, 396994 Uka Tarsadia University, Gopal Vidyanagar, Bardoli-Mahuva Road, Tarsadi, Gujarat 394350, India; ‡ Parul Institute of Pharmacy and Research, Faculty of Pharmacy, 284499 Parul University, P.O. Limda, Waghodia, Vadodara, Gujarat 391760, India; § Department of Eye and Vision Science, Institute of Life Course and Medical Sciences, 4591 University of Liverpool, 6 West Derby Street, Liverpool L7 8TX, United Kingdom; ∥ School of Pharmacy, 1596 Queen’s University Belfast, 97 Lisburn Road, Belfast BT9 7BL, United Kingdom

## Abstract

Prostate cancer is the second most common cancer in men worldwide, highlighting the urgent need for effective and targeted chemotherapeutic approaches. This study reports the development and optimization of 5-fluorouracil (5-FU)–loaded poly­(lactic-*co*-glycolic acid)–polyethylene glycol–folic acid (PLGA–PEG–FOL) nanoparticles designed for folate receptor–mediated targeted therapy. The PLGA–PEG–FOL conjugate was synthesized via a stepwise carbodiimide coupling reaction and confirmed by FT-IR analysis. Nanoparticles were formulated via a modified emulsification–solvent evaporation method and optimized through a Box–Behnken design. The optimized formulation demonstrated a particle size of 178.47 ±  3.26 nm, a narrow polydispersity index (0.119 ±  0.008), a zeta potential of −23.4  ± 0.35 mV, a high entrapment efficiency (78.93 ±  1.05%), and sustained release of 5-FU for up to 72 h. In vitro cytotoxicity assays in PC-3 prostate cancer cells revealed a 1.6-fold reduction in the IC_50_ value compared with that of free 5-FU, indicating enhanced therapeutic potency. In vivo efficacy was evaluated in testosterone-induced prostate cancer in male Wistar rats. Compared with the control, treatment with 5-FU-loaded PLGA–PEG–FOL nanoparticles significantly reduced the prostate index and produced a 2.2-fold decrease in serum PSA levels and a 1.9-fold decrease in serum testosterone levels. Histopathological examination confirmed the attenuation of hyperplastic and dysplastic lesions in the nanoparticle-treated group. These findings suggest that PLGA–PEG–FOL nanoparticles are a promising targeted delivery platform for enhancing the therapeutic efficacy of 5-FU in prostate cancer treatment.

## Graphical abstract

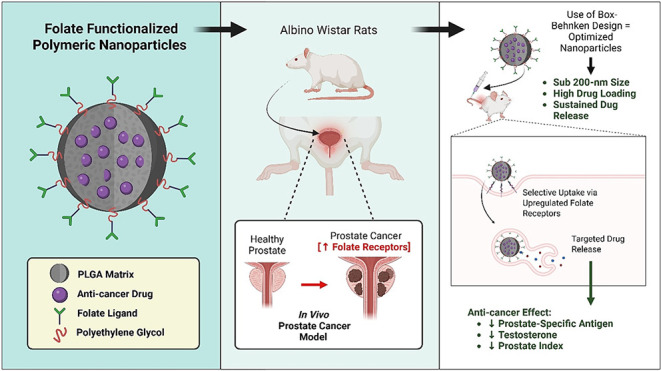

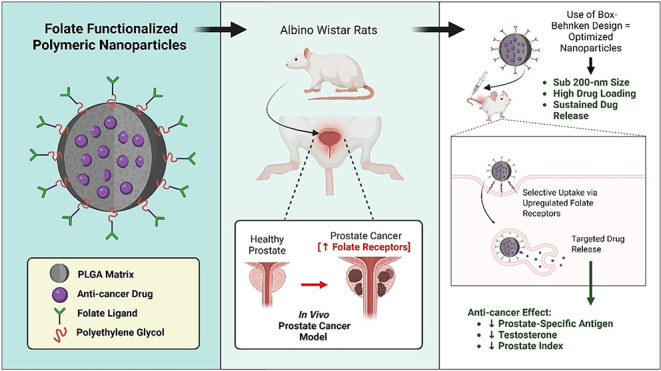

## 1 Introduction

Prostate cancer remains a formidable global health challenge, with its incidence continuing to rise owing to increasing life expectancy, lifestyle transitions, and enhanced screening strategies.^1^ According to the most recent estimates from GLOBOCAN 2022, prostate cancer is the second most commonly diagnosed cancer in men worldwide, accounting for approximately 1.47 million new cases, surpassed only by lung cancer in terms of incidence among males.^2^ While localized prostate cancer can often be managed effectively with surgery or radiotherapy, treatment options for advanced or metastatic prostate cancer are far more limited, with systemic chemotherapy, hormonal therapy, or androgen deprivation therapy (ADT) offering only temporary disease control.^3,4^ One of the key chemotherapeutic agents used in prostate cancer and other solid tumors is 5-fluorouracil (5-FU), a pyrimidine analogue that disrupts DNA synthesis by inhibiting thymidylate synthase.^5^ Despite its longstanding clinical relevance, the therapeutic utility of 5-FU is hampered by rapid systemic clearance, a short plasma half-life, poor tumor selectivity, and severe dose-limiting toxicities such as myelosuppression and mucositis.^6–9^ These pharmacokinetic limitations underscore the urgent need for targeted and sustained-release delivery systems capable of enhancing tumor accumulation while minimizing systemic exposure.^10^

Polymeric nanoparticles, particularly those based on poly­(lactic-*co*-glycolic acid) (PLGA), have garnered significant attention for cancer drug delivery because of their biodegradability, biocompatibility, and regulatory approval.^11,12^ When combined with polyethylene glycol (PEG), PLGA nanoparticles exhibit improved colloidal stability, reduced recognition by the reticuloendothelial system (RES), and prolonged systemic circulation.^13–16^ Additionally, surface functionalization with targeting ligands such as folic acid (FOL) allows active targeting via folate receptors (FRs), which are overexpressed in a variety of cancers, including prostate cancer, but are largely absent in normal tissues.^17–20^ This dual-targeting strategy, which involves passive targeting via the enhanced permeability and retention (EPR) effect and active targeting via receptor-mediated endocytosis, has shown promise in enhancing the therapeutic index and reducing off-target toxicity.^21^

Beyond folate functionalization, numerous ligand-mediated targeting strategies have been investigated to improve drug delivery in prostate cancer. These include prostate-specific membrane antigen-targeted nanoparticles,^22,23^ transferrin-modified carriers,^24,25^ hyaluronic acid-decorated systems,^26,27^ and antibody-conjugated nanocarriers,^28,29^ all designed to improve tumor-specific uptake and reduce systemic toxicity. A wide range of payloads (such as docetaxel, paclitaxel, doxorubicin, curcumin, and small interfering RNA) has been successfully encapsulated within such systems, demonstrating improved pharmacokinetics and therapeutic outcomes in preclinical prostate cancer models.^30–34^ These studies collectively highlight the potential of receptor-targeted nanocarriers in prostate cancer therapy while underscoring the need for improved formulation strategies that ensure stability, sustained release, and *in vivo* validation.

Previous studies have explored folate-functionalized PLGA nanoparticles for the delivery of 5-FU and other anticancer drugs and demonstrated enhanced in vitro cytotoxicity and cellular uptake.^35,36^ However, many of these systems lacked PEGylation, which is essential for improving systemic stability, reducing opsonization, and extending circulation time. Furthermore, most did not employ statistical design tools to systematically optimize formulation variables, limiting the reproducibility and translational reliability of the results. Only a few studies have reported PLGA–PEG–FOL nanoparticles for prostate cancer, and these largely involved other therapeutic payloads such as docetaxel and resveratrol, with minimal integration of pharmacodynamic or histopathological validation.^37,38^

To address these gaps, we developed a systematically optimized folate-functionalized PLGA–PEG nanoparticle system for 5-FU delivery that is specifically tailored for prostate cancer therapy. Employing a Box–Behnken design, we engineered nanoparticles with high entrapment efficiency, uniform size distribution, and sustained release kinetics. The formulation was rigorously characterized for its physicochemical properties, morphology, and folate conjugation efficiency. In vitro studies confirmed cytotoxicity in PC-3 prostate cancer cells, whereas in vivo evaluation utilized a translational testosterone-induced prostate hyperplasia rat model, a physiologically relevant system mimicking androgen-driven tumorigenesis. Therapeutic efficacy was assessed through prostate-specific antigen (PSA) and testosterone levels, alongside histopathological validation of tumor regression. The novelty of this study lies not in the synthetic chemistry of PLGA–PEG–FOL itself but in its integrated application for 5-FU delivery, combining systematic design-of-experiments optimization with comprehensive in vitro and in vivo prostate cancer evaluation. By combining active folate receptor targeting, PEGylation-driven therapeutic enhancement, and design-of-experiments-guided optimization, this work provides a novel, clinically relevant framework for repurposing 5-FU via a nanocarrier platform tailored for prostate cancer therapy.

## 2 Materials

Poly­(d,l-lactide-*co*-glycolide) (PLGA, 50:50, carboxyl-terminated, inherent viscosity of 0.20 dL/g) was generously provided by Nomisma Healthcare Pvt. Ltd. (Vadodara, India). Bis-amine polyethylene glycol (NH_2_–PEG–NH_2_, molecular weight ∼4000 Da; H_2_N–CH_2_CH_2_–O–(CH_2_CH_2_O)_n_–CH_2_CH_2_–NH_2_, *n* ≈ 90) and folic acid were obtained from Yarrow Chem Products (Mumbai, India). 5-Fluorouracil (5-FU), *N*,*N*′-dicyclohexylcarbodiimide (DCC), *N*-hydroxysuccinimide (NHS), dichloromethane (DCM), methanol, acetone, dimethyl sulfoxide (DMSO), diethyl ether, sodium carbonate, and testosterone were purchased from Fine-Chem Ltd. (Mumbai, India). Poloxamer 188 (Pluronic F68) was sourced from Lubrizol India Pvt. Ltd. (Mumbai, India) and used as a stabilizing agent in nanoparticle formulations. Cell culture reagents, including RPMI-1640 medium, fetal bovine serum (FBS), and antibiotic–antimycotic solution, were procured from HiMedia Laboratories Pvt. Ltd. (Mumbai, India). All chemicals and reagents were of analytical or cell culture grade and were used as received without further purification.

## 3 Methods

### 3.1 Analytical Methods

The quantitative estimation of 5-FU in both the mobile phase and plasma samples was performed via a high-performance liquid chromatography (HPLC) system (Jasco LC-4000 series, Easton, MD, USA) equipped with an integrated degasser, binary pump, automatic sample injector, and MD 4010 UV–visible detector. Chromatographic separation was achieved on a Phenomenex C18 column (250 × 4.6 mm, 5 μm particle size). The column temperature was maintained at 25 ± 2 °C. The mobile phase consisted of 5 mmol/L potassium dihydrogen phosphate buffer (adjusted to pH 6.0) and methanol in a 95:5 (v/v) ratio. The flow rate was maintained at 1.0 mL/min, and the eluent was monitored at a wavelength of 254 nm.^39,40^ The injection volume was 20 μL for all of the samples. This method provided reproducible retention times and baseline separation of 5-FU under the described conditions.

### 3.2 Synthesis of the PLGA–PEG–FOL Conjugates

The PLGA–PEG–FOL conjugate was synthesized via a sequential three-step process involving the activation of PLGA, PEGylation, and subsequent folic acid conjugation.^41–43^ Initially, PLGA was activated by reacting with N,N′-dicyclohexylcarbodiimide (DCC) and *N*-hydroxysuccinimide (NHS) in dichloromethane (DCM) at a molar ratio of 1:5:5 (PLGA:DCC:NHS) under a nitrogen atmosphere at room temperature overnight. The resulting solution was filtered (Whatman grade 1) to remove insoluble byproducts.^44^ In the second step, the activated PLGA was reacted with bis-amine polyethylene glycol under identical inert conditions to obtain the PLGA–PEG intermediate. The primary amine group of NH_2_–PEG–NH_2_ participated in conjugation with the activated carboxylic group of PLGA via amide bond formation. The PLGA–PEG intermediate was not characterized separately, as this conjugate has been extensively reported in the literature, and the reaction conditions were carefully controlled to minimize side reactions. To prevent potential cross-linking caused by both terminal amine groups of PEG, PEG was used in excess relative to PLGA during the PEGylation step. The crude product was purified by three cycles of precipitation in an ice-cold methanol:diethyl ether mixture (1:1, v/v), centrifugation at 5000 × g for 10 min, and resuspension in DCM, followed by vacuum drying at 25 ± 0.5 °C using a REMI RDHO-50 dryer.^45^ In the final step, the dried PLGA–PEG intermediate was dissolved in dimethyl sulfoxide (DMSO), and folic acid (FOL) was activated with DCC and NHS at a molar ratio of 1:5:5 (FOL:DCC:NHS) before conjugation with PLGA–PEG under a nitrogen atmosphere at room temperature overnight. The reaction mixture was filtered and dialyzed against 5 mM sodium carbonate buffer (pH 8.0) using a Spectra/Por membrane (MWCO 3500) for 8 h with buffer replacement every 4 h, followed by overnight dialysis in deionized water (20 mL per cycle) to remove unreacted reagents and byproducts.^46^ The resulting PLGA–PEG–FOL conjugate was freeze-dried via a benchtop lyophilizer (Labconco) and stored for further use.

### 3.3 FT-IR Characterization

Fourier transform infrared (FT-IR) spectra were recorded by using a Bruker α spectrometer (Japan) equipped with an attenuated total reflectance (ATR) accessory. Spectral analysis was performed for pure PLGA, PEG, FOL, and the synthesized PLGA–PEG–FOL conjugate to identify characteristic functional groups and confirm successful conjugation. Approximately 2 mg of each dried sample was directly placed on the diamond ATR crystal, and spectra were collected over the range of 4000–500 cm^–1^ at ambient temperature. As ATR-FTIR does not require precise concentration control, all spectra were acquired under identical conditions (32 scans, 4 cm^–1^ resolution) to ensure consistency across samples. The resulting spectra were analyzed to verify the presence of specific chemical functionalities and molecular interactions indicative of polymer conjugation and drug encapsulation.

### 3.4 Formulation of PLGA–PEG–FOL Nanoparticles

PLGA–PEG–FOL nanoparticles were prepared via a modified emulsification–solvent evaporation technique.^47,48^ The organic phase was prepared by dissolving 5-FU and the synthesized PLGA–PEG–FOL conjugate in acetone, while the aqueous phase comprised a 1.5% (w/v) solution of Poloxamer 188, which served as a stabilizer. Under continuous magnetic stirring, the organic phase was added dropwise to the aqueous phase at a controlled flow rate of 0.3 mL/min, and stirring was continued for 15 min. The resulting emulsion was subjected to solvent evaporation via a rotary evaporator (Rotary Evaporator R-100, Büchi) to yield an aqueous nanoparticle dispersion. The dispersion was centrifuged at 20,000 rpm (≈35,800*g*) for 60 min at 5 °C (C-24 Plus Cooling Centrifuge, REMI), followed by three washing cycles to remove unencapsulated drug and residual polymer.^49^ Drug-free (blank) nanoparticles were prepared via the same method without the addition of 5-FU.

The formulation parameters were optimized via a Box–Behnken design, where the aqueous-to-organic phase ratio (*X*_1_), polymer-to-drug ratio (*X*_2_), and stabilizer concentration (*X*_3_) were selected as independent variables.^50,51^ The upper and lower limits of these variables were determined based on preliminary optimization trials conducted in our laboratory to ensure feasible formulation and stable nanoparticle formation. These ranges also fall within the concentration limits reported by several other research groups working on similar PLGA-based nanoparticle systems.^52–54^ The BBD was chosen because it requires fewer experimental runs compared with other designs, efficiently explores quadratic response surfaces, and avoids extreme factor combinations that may result in unstable formulations. This makes it particularly suitable for nanoparticle optimization, where experimental resources are limited, and formulation stability is a key consideration. The corresponding responses were particle size (*Y*_1_), entrapment efficiency (*Y*_2_), and time to 80% drug release (*T*_80_, *Y*_3_). *T*_80_ (time required for 80% drug release) was selected as the response variable because it provides a reliable measure of sustained-release performance, representing the point at which most of the encapsulated drug is released while the extended-release characteristics of the formulation. It offers better differentiation among formulations compared to earlier release endpoints such as T_50_ or % release at fixed time intervals.

A total of 15 experimental runs, including center points, were generated and evaluated via Design Expert software (Version 13, Stat-Ease, Inc., Minneapolis, USA). Polynomial regression models were developed to assess the main interactive and quadratic effects of formulation factors on the responses. Model selection was based on statistical criteria, including *R* ^2^, adjusted *R* ^2^, and predicted *R* ^2^ values. Model validity was confirmed by comparing the predicted and experimental results from the checkpoint batches. Response surface methodology was used to generate three-dimensional plots illustrating the influence of formulation variables.^55,56^ The formulation was optimized via a combination of numerical and graphical optimization techniques, applying predefined constraints: a particle size below 200 nm, an entrapment efficiency exceeding 70%, and a target *T*_80_ (time required for 80% drug release) of approximately 48 h.^57^ Design Expert software generates multiple formulation solutions, each of which is evaluated on the basis of a calculated desirability index. The optimized batch was selected because it exhibited the highest overall desirability and was subsequently prepared via the formulation procedure described earlier.

### 3.5 Characterization of PLGA–PEG–FOL Nanoparticles

#### 3.5.1 Particle Size, Polydispersity Index, and Zeta Potential

The 5-FU-loaded PLGA–PEG–FOL nanoparticles were characterized for their particle size, polydispersity index (PDI), and zeta potential via a Zetasizer Nano-ZS90 (Malvern Instruments, UK) via dynamic light scattering (DLS) at a fixed detection angle of 90°.^58^ The samples were diluted 100-fold (v/v) with ultrapure water to ensure unrestricted Brownian motion and minimize multiple scattering effects.^59^ Measurements were conducted in triplicate at 25.0 ±  0.1 °C, with 20 s intervals between runs. Zeta potential and electrophoretic mobility were calculated via Malvern’s Dispersion Technology Software. All of the results are reported as the means  ±  standard deviations (SDs).

#### 3.5.2 Entrapment Efficiency and Drug Loading

To determine the entrapment efficiency and drug loading, the 5-FU-loaded PLGA–PEG–FOL nanoparticles were centrifuged at 20,000 rpm (≈35,800*g*) for 10 min at 4 °C in a refrigerated centrifuge (REMI C24 Plus, India). The supernatant was collected, and the amount of unencapsulated (free) 5-FU was quantified via the HPLC method described previously.^60,61^ The entrapment efficiency (EE%) and drug loading (DL%) were calculated via the following equations:

$$EE\%=\frac{Initial\;amount\;of\,\;drug-Free\;drug\;in\;superna\tan t}{Initial\;amount\;of\;drug}\times 100$$

$$\mathrm{DL}\%=\frac{\mathrm{Initial}\;\mathrm{amount}\;\mathrm{of}\;\mathrm{drug}-\mathrm{Free}\;\mathrm{drug}\;\mathrm{in}\;\mathrm{supernatant}}{\mathrm{Total}\;\mathrm{weight}\;\mathrm{of}\;\mathrm{nanoparticles}}\times 100$$

#### 3.5.3 Drug Release Study

The in vitro release profile of 5-FU from PLGA–PEG–FOL nanoparticles was assessed via Franz diffusion cell assembly with a dialysis membrane (Spectra/Por, molecular weight cutoff of 12,000–14,000 Da). Prior to use, the membrane was presoaked in distilled water at room temperature for 12 h to ensure hydration and flexibility. A 2 mL aliquot of nanoparticle suspension, corresponding to an equivalent 5-FU concentration of 10 mg/mL, was placed in the donor compartment, while the receptor compartment was filled with 50 mL of phosphate-buffered saline (PBS, pH 7.4) to maintain physiological conditions. The system was maintained at 37.0  ±  0.5 °C and stirred continuously at 100 rpm throughout the experiment to simulate in vivo sink conditions.^62,63^ At predetermined time intervals (0.25, 0.5, 1, 2, 3, 6, 9, 12, 24, 48, and 52 h), 1 mL of receptor medium was withdrawn and immediately replaced with an equal volume of fresh PBS to maintain a constant volume and sustained sink environment.^64^ The concentration of 5-FU in the collected samples was determined via the validated HPLC method described earlier. All release experiments were performed in triplicate, and the results are expressed as the cumulative percentages of release over time.

#### 3.5.4 Lyophilization of the Nanoparticles

The optimized 5-FU-loaded PLGA–PEG–FOL nanoparticles were lyophilized via a benchtop freeze-dryer (FreeZone 2.5-L, Labconco, Mumbai, India) to increase their stability and facilitate long-term storage.^65^ For each batch, 2 mL of nanoparticle dispersion was mixed with 2 mL of a 3% w/v mannitol solution, which served as a cryoprotectant. The mixture was transferred to lyophilization vials and subjected to a controlled freeze–drying cycle. Initially, the samples were frozen at −50 °C for 48 h, followed by primary drying at −30 °C under a vacuum of 150 mTorr for 24 h. The process was completed with secondary drying at 22 °C and 50 mTorr for 6 h.^66^ The resulting lyophilized nanopowder was stored in airtight containers under desiccated conditions until further analysis.

#### 3.5.5 Scanning and Transmission Electron Microscopy

The surface morphology and internal structure of the optimized lyophilized 5-FU-loaded PLGA–PEG–FOL nanoparticles were characterized by using scanning electron microscopy (SEM) and transmission electron microscopy (TEM). For SEM analysis, 10 mg of the freeze-dried nanoparticles were dispersed in 1 mL of deionized water, and 5 μL of this suspension was drop-cast onto a clean glass slide. The samples were dried in a vacuum oven to ensure complete solvent removal.^67,68^ Subsequently, the dried films were gold-coated using a Polaron sputter coater (Polaron E5100, Polaron Equipment Ltd., Hertfordshire, UK). The coated samples were imaged using a field-emission scanning electron microscope (Gemini, Zeiss, Oberkochen, Germany) operated at an accelerating voltage of 5 kV and magnifications up to 100,000× to examine nanoparticle morphology.^69^ For TEM analysis, 30 μL of the nanoparticle suspension (2 mg/mL in deionized water) was diluted with 4 mL of distilled water. A 0.5 μL aliquot of this diluted suspension was drop-cast onto a carbon-coated copper grid (200 mesh) and dried for 10 min under an infrared lamp. The grid was then negatively stained with 1% phosphotungstic acid for 20 s, and excess stain was removed with filter paper. Imaging was performed using a Tecnai G2 Spirit BioTwin transmission electron microscope (FEI, The Netherlands) operated at 120 kV, equipped with an Olympus VELETA CCD camera, to visualize particle shape and surface smoothness at the nanoscale at a magnification of 100,000×.^44^

### 3.6 Cell Line Studies

#### 3.6.1 Cell Culture

The human prostate cancer cell line PC-3 (ATCC CRL-1435), obtained from the National Centre for Cell Sciences (NCCS, Pune, India), was used for in vitro experiments. The cells were cultured in Dulbecco’s modified Eagle’s medium with high glucose (DMEM-HG) supplemented with 10% fetal bovine serum (FBS) and 1% antibiotic solution containing 100 μg/mL streptomycin and 100 U/mL penicillin. Cultures were maintained at 37 °C in a humidified incubator with 5% CO_2_. Upon reaching approximately 80% confluency, the medium was replaced with fresh complete medium for subsequent experiments.

#### 3.6.2 In Vitro Cytotoxicity Assay

PC-3 human prostate cancer cells were seeded at a density of 20,000 cells per well in 96-well plates and incubated overnight to allow proper attachment. Four treatment groups were investigated across a concentration range of 4 to 1024 μg/mL: PLGA–PEG nanoparticles (Blank) to assess carrier compatibility and to exclude any cytotoxic effect of PEGylation or folate chemistry; 5-FU solution to serve as the reference drug; PLGA–PEG nanoparticles loaded with 5-FU to evaluate the impact of encapsulation; and PLGA–PEG–FOL nanoparticles loaded with 5-FU as the main targeted formulation. To ensure a fair comparison, blank PLGA–PEG nanoparticles were applied at polymer concentrations equivalent to those present in the corresponding drug-loaded formulations. This allowed us to confirm the intrinsic cytocompatibility of the carrier system across the full concentration range and ensure that any cytotoxicity observed in the drug-loaded groups was attributable to 5-FU rather than the nanoparticle matrix.^70^ After 48 h of treatment, the medium was discarded, cells were rinsed with phosphate-buffered saline, and 200 μL of MTT reagent (0.5 mg/mL in serum-free medium) was added to each well. Plates were incubated for 3 h, the reagent was removed, and 100 μL of DMSO was added to dissolve the formazan crystals. Absorbance was recorded at 540 nm with background correction at 690 nm using a microplate reader (Model 680, Bio-Rad Laboratories, Hercules, CA, USA).^71,72^ Cell viability was expressed as the percentage of treated cells relative to the untreated controls. All experiments were performed in triplicate with three independent repeats. Concentration–response curves were generated using a four-parameter logistic model, and IC_50_ was calculated by nonlinear regression. Statistical differences were evaluated using one-way ANOVA followed by appropriate multiple comparison tests, with significance set at *p* < 0.05.

### 3.7 In Vivo Anticancer Studies

#### 3.7.1 Ethical Approval and Animal Housing

All animal procedures were reviewed and approved by the Institutional Animal Ethical Committee (IAEC) of Maliba Pharmacy College, Bardoli, India, and conducted in accordance with the guidelines established by the Committee for the Purpose of Control and Supervision of Experiments on Animals (CPCSEA), Government of India (Protocol No. MPC/IAEC/10/2020). Male albino Wistar rats (250 ± 20 g, age: 8–10 weeks) were procured from the Central Animal House of Maliba Pharmacy College. The animals were housed under standard laboratory conditions at a controlled temperature of 25  ±  2 °C with a 12-h light/dark cycle and had free access to a standard pellet diet and water throughout the study period.

#### 3.7.2 Prostate Cancer Induction and Treatment Protocol

PC was induced in male albino Wistar rats via the subcutaneous administration of exogenous testosterone, a known promoter of prostatic adenocarcinogenesis, particularly in the dorsolateral and anterior lobes of the prostate.^73^ The study animals were initially divided into two groups: Group I (*n* = 8), which served as the negative control, received 1 mL/kg arachis oil subcutaneously once daily for 28 consecutive days; Group II (*n* = 26), designated for PC induction, received 3 mg/kg testosterone (dissolved in arachis oil) subcutaneously once daily for the same duration. At the end of the induction period, testosterone administration was discontinued, and animals from Group II and Group I were sacrificed to confirm successful prostate cancer induction via histopathological examination. The remaining 24 rats from Group II were randomly assigned to three treatment groups (*n* = 8 per group): Group A (disease control) received 2 mL of distilled water orally for 14 days; Group B received 15 mg/kg optimized 5-FU-loaded PLGA–PEG–FOL nanoparticles orally for 14 days; and Group C received 15 mg/kg plain 5-FU solution intraperitoneally for the same duration. The oral route was deliberately selected for the PLGA–PEG–FOL nanoparticles to evaluate their capability to overcome the inherent pharmacokinetic limitations of 5-FU. Conventional 5-FU exhibits extremely poor oral bioavailability due to extensive first-pass metabolism by dihydropyrimidine dehydrogenase, which catabolises up to 80–90% of the administered dose before systemic exposure.^74,75^ The nanoparticle formulation was therefore designed to provide sustained release, improved mucosal absorption, and partial protection of the drug from enzymatic degradation, thereby enabling a potential oral delivery. In contrast, the 5-FU solution was administered intraperitoneally as a standard reference, representing the conventional parenteral route used clinically for 5-FU chemotherapy. This design allows comparative evaluation of therapeutic efficacy between conventional systemic administration and the novel oral nanoparticle system while acknowledging that pharmacokinetic differences between the two routes are an inherent limitation of this preliminary in vivo study.

Following the treatment phase, all of the animals were anaesthetized with diethyl ether and sacrificed. Prostate tissues were collected for evaluation of the prostate index (prostate weight/body weight), while blood samples were analyzed for serum prostate-specific antigen (PSA) and testosterone levels. Additionally, histopathological assessments of the prostate were conducted to evaluate therapeutic outcomes and compare the in vivo anticancer efficacy of the nanoparticle formulation with that of free 5-FU and untreated controls.^76,77^

##### 3.7.2.1 Prostate Index Evaluation

The prostate index serves as a physiological marker for assessing treatment-related effects on prostate size and is commonly used as an indirect indicator of therapeutic outcomes in prostate cancer models.^78^ At the end of the treatment period, the animals were euthanized and the prostate glands were carefully excised and weighed. The total body weight was also recorded prior to sacrifice. The prostate index (PI) for each animal was calculated by dividing the prostate weight by the corresponding total body weight. This normalization allowed for accurate comparisons of prostate enlargement and regression across different treatment groups.

##### 3.7.2.2 Serum Biomarker Analysis

At the end of the treatment period, blood samples were collected from the retro-orbital plexus under light anesthesia. The samples were allowed to clot at room temperature for 20 min and subsequently centrifuged at 3000 rpm (≈805*g*) for 10 min to isolate the serum. Serum levels of prostate-specific antigen (PSA) and testosterone were quantified via commercially available enzyme-linked immunosorbent assay (ELISA) kits.^79,80^ PSA levels were determined via a kit from Rapid Laboratories Ltd. (Colchester, Essex, U.K.), whereas testosterone concentrations were measured via ELISA kits procured from MP Biomedicals (Ohio, USA), both of which were performed according to the manufacturers’ protocols. All of the assays were conducted in triplicate, and the results are expressed as nanograms per mL (ng/mL).

##### 3.7.2.3 Histopathological Evaluation

Prostate tissues were excised from euthanized rats and immediately flushed with normal saline, followed by fixation in 10% neutral buffered formalin for 24 h. The fixed tissues were dehydrated through a graded ethanol series, cleared with benzene, and embedded in paraffin blocks. Serial sections of 5 μm thickness were prepared via a microtome. The paraffin-embedded sections were deparaffinized with xylene and rehydrated through descending grades of ethanol. After being rinsed with phosphate-buffered saline (PBS), the sections were permeabilized with a solution of 0.1 M citrate and 0.1% Triton X-100. Hematoxylin and eosin (H&E) staining was performed to visualize the cellular and tissue architecture. The stained sections were examined under a light microscope at 40× magnification to assess histological alterations in prostate morphology across treatment groups.

### 3.8 Statistical Analysis

All of the results are expressed as the means ± standard deviations (SDs). Prior to inferential testing, the data were assessed for the normality and homogeneity of variance. Statistical comparisons between two groups were performed via two-tailed Student’s *t* test, whereas comparisons among multiple groups were evaluated via one-way analysis of variance (ANOVA), followed by appropriate post hoc testing where applicable. A *P* value ≤0.05 was considered statistically significant. All of the statistical analyses were conducted via GraphPad Prism (GraphPad Software, Inc.) and SPSS (IBM SPSS Statistics, Chicago, IL, USA).

## 4 Results and Discussion

### 4.1 FT-IR Analysis of the PLGA–PEG–FOL Conjugates

The PLGA–PEG–FOL conjugate was successfully synthesized through a three-step conjugation process (Scheme 1). In the first step, the terminal carboxylic acid group of PLGA was activated by using DCC and NHS and covalently coupled to the primary amine group of PEG, forming the intermediate PLGA–PEG copolymer. In the second step, the carboxyl group of FOL was similarly activated and conjugated to the terminal amine of the PLGA–PEG intermediate, yielding the final PLGA–PEG–FOL conjugate. To confirm the presence of amide linkages within the conjugated polymer, FT-IR spectroscopy was performed (Figure 1). In both the control sample and the functionalized copolymer, characteristic absorption peaks for folate were observed at 1419 and 1607 cm^–1^, which correspond to the stretching vibrations of the C=C bond in the aromatic ring of folic acid. A prominent FT-IR absorption peak specific to the PLGA–PEG–FOL conjugate was identified at 1627 and 1570 cm^–1^, which are indicative of the carbonyl (C=O) and amine (N–H) stretching vibrations, respectively, in the amide linkages formed between PLGA–PEG and folic acid. These amide bands were absent in the control sample, confirming successful conjugation. The presence of these characteristic peaks is consistent with previous reports on similar polymer modifications, further validating the formation of the PLGA–PEG–FOL conjugate.^81–83^

**Figure 1:**
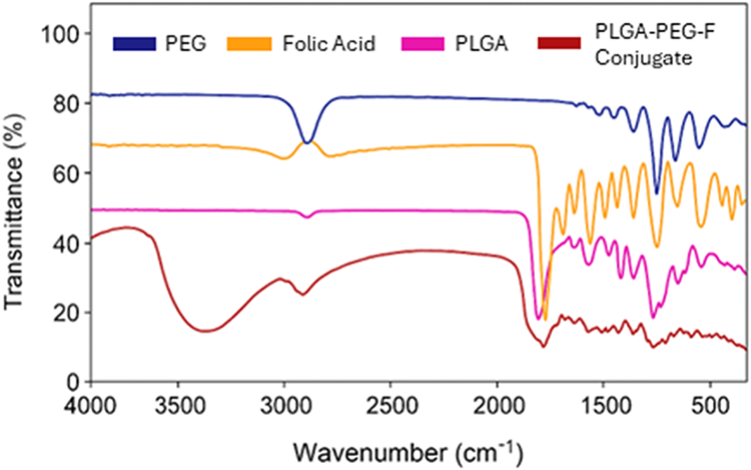
FT-IR spectral comparison of individual components (PLGA, PEG, and folic acid) and the synthesized PLGA–PEG–FOL conjugate. Characteristic absorption bands confirming successful conjugation include amide bond–specific peaks (∼1627 cm^–1^ for C=O and ∼1570 cm^–1^ for N–H stretching), which are present in the final conjugate but absent in the individual precursors, confirming the formation of amide linkages between PLGA, PEG, and FOL.

**Scheme 1:**
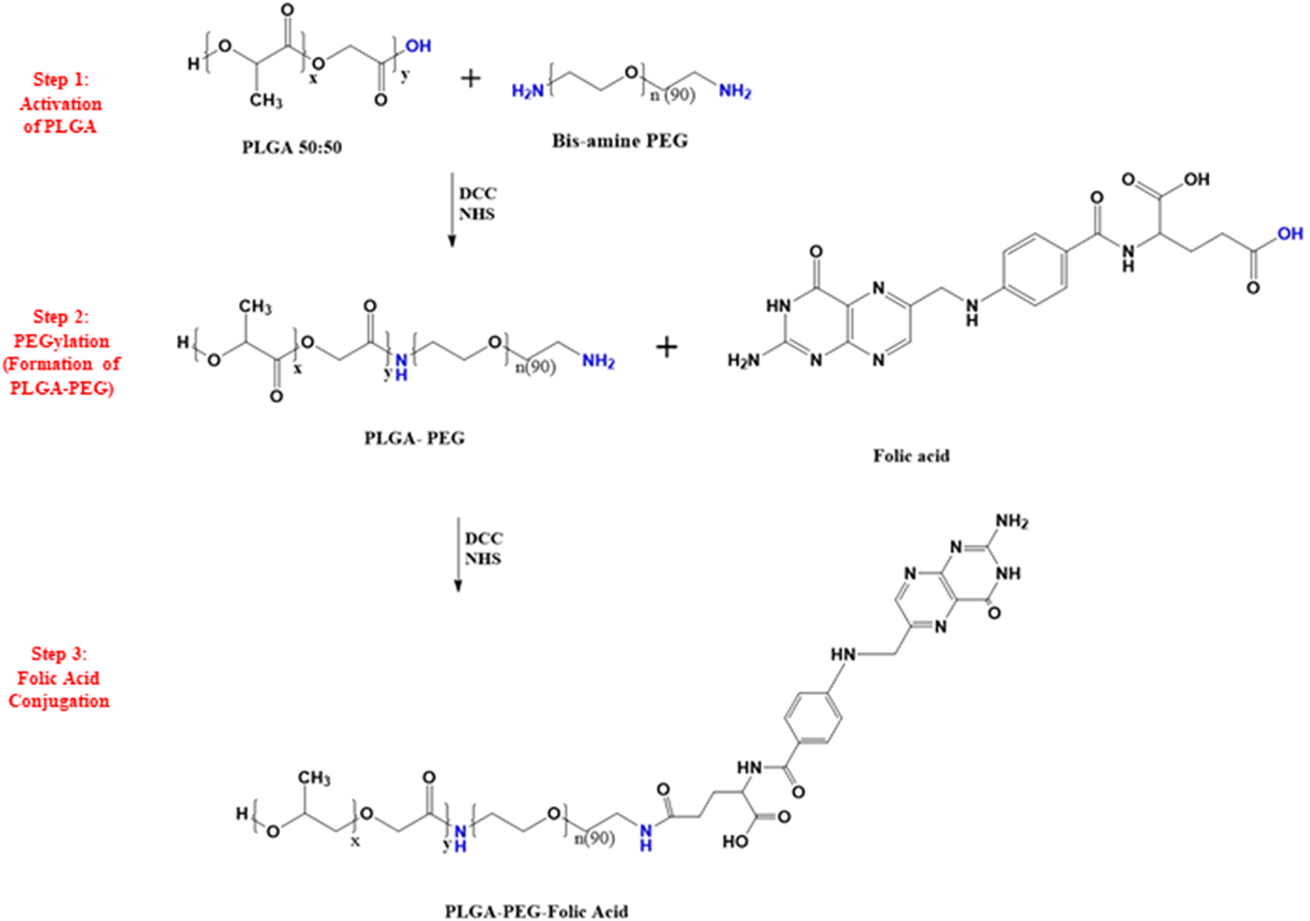
Stepwise Synthesis of the PLGA–PEG–FOL Conjugate^a^

a PLGA represents poly­(lactic-*co*-glycolic acid) with repeating units *x*/*y* = 50:50. The bis-amine polyethylene glycol used in the PEGylation step had an average repeating unit number *n* ≈ 90, and the PEG sample was polydispersed.

### 4.2 Formulation Optimization via the Box–Behnken Design

The optimization of 5-FU-loaded PLGA–PEG–FOL nanoparticles was carried out via a Box–Behnken statistical design to systematically evaluate the influence of three independent formulation variables: the aqueous phase–organic phase ratio (*X*_1_), the polymer–drug ratio (*X*_2_), and the stabilizer concentration (%) (*X*_3_). The response variables assessed were particle size (*Y*_1_), entrapment efficiency (*Y*_2_), and *T*_80_ (the time required to release 80% of the drug) (*Y*_3_). The experimental matrix and observed results are summarized in Table 1.

**Table 1:** Box–Behnken Design Matrix for the Optimization of Independent Variables^a^

Batch no.	*X*_1_	*X*_2_	*X*_3_	*Y*_1_ (nm) ± SD	*Y*_2_ (%) ± SD	*Y*_3_ (h) ± SD
F1	4.00	8.00	1.50	215.3 ± 5.6	80.5 ± 3.4	49.2 ± 1.6
F2	2.00	4.00	1.50	210.1 ± 4.2	79.2 ± 4.1	52.7 ± 2.6
F3	2.00	8.00	1.50	291.8 ± 10.6	89.4 ± 3.9	70.3 ± 3.5
F4	4.00	4.00	1.50	179.6 ± 5.2	67.3 ± 2.8	51.5 ± 1.1
F5	3.00	4.00	0.50	219.3 ± 7.9	75.2 ± 5.1	53.3 ± 2.7
F6	3.00	6.00	1.50	188.4 ± 8.1	73.7 ± 2.1	47.6 ± 1.4
F7	2.00	6.00	2.50	268.6 ± 9.7	85.1 ± 2.6	64.9 ± 2.1
F8	4.00	6.00	0.50	274.7 ± 4.5	82.6 ± 6.0	55.9 ± 1.9
F9	3.00	6.00	1.50	197.5 ± 7.1	72.1 ± 3.8	48.3 ± 2.2
F10	4.00	6.00	2.50	178.3 ± 6.7	71.0 ± 4.8	46.8 ± 1.8
F11	3.00	4.00	2.50	185.1 ± 4.5	73.7 ± 5.2	48.4 ± 2.6
F12	3.00	6.00	1.50	193.0 ± 3.1	75.4 ± 5.6	50.2 ± 2.6
F13	3.00	8.00	0.50	297.1 ± 11.2	88.6 ± 3.1	63.1 ± 3.1
F14	2.00	6.00	0.50	294.8 ± 8.1	85.9 ± 2.3	65.2 ± 2.2
F15	3.00	8.00	2.50	202.5 ± 7.5	78.9 ± 4.5	56.8 ± 2.4

a Here, *X*_1_ – Aqueous phase:organic phase ratio, *X*_2_ – Polymer:drug ratio, *X*_3_ – stabilizer concentration (%), *Y*_1_ – particle size (nm), *Y*_2_ – entrapment efficiency (%) and *Y*_3_. *T*_80_ – time for 80% drug release in h.

Regression analysis confirmed that a quadratic model best fit each response variable, as indicated by high *F* values and strong correlation coefficients (*R* ^2^, adjusted *R* ^2^, and predicted *R* ^2^), all of which exceeded 0.86 with desirable adequate precision (Table 2). These metrics validated the robustness and predictive reliability of the models.^84,85^

**Table 2:** Statistical Parameters for the Developed Models

Response Variable	Selected Model	Model *F* value	*R* ^2^	Adjusted *R* ^2^	Predicted *R* ^2^	Adequate Precision
Particle size	Quadratic	145.29	0.9962	0.9893	0.9589	31.741
Entrapment efficiency	Quadratic	34.80	0.9842	0.9558	0.8661	18.252
*T*_80_	Quadratic	52.37	0.9895	0.9706	0.8948	22.859

The particle size of the formulated NPs ranged from 176 to 297 nm. The analysis revealed that increasing the aqueous phase:organic phase ratio led to a significant reduction in the particle size. This behavior is attributed to enhanced solvent diffusion and the formation of finer emulsified globules during nanoprecipitation, facilitated by a higher aqueous content.^86,87^ Conversely, increasing the polymer:drug ratio resulted in larger nanoparticles, likely due to increased internal phase viscosity impeding uniform dispersion. The stabilizer concentration exhibited an inverse relationship with particle size, supporting its critical role in stabilizing smaller particles through efficient emulsification.^88^

The polynomial model for the particle size was as follows:

$$Y_{1}=192.97-27.18X_{1}+26.57X_{2}-31.43X_{3}-11.50X_{1}X_{2}-17.55X_{1}X_{3}-15.10X_{2}X_{3}+29.67{X_{1}}^{2}+31.47{X_{3}}^{2}$$

The entrapment efficiency ranged from 67.3 to 89.4%. This ratio increased with increasing polymer:drug ratios, indicating that more polymer was available to encapsulate the drug. However, the greater aqueous phase and stabilizer concentrations negatively influence entrapment. A higher stabilizer concentration may accelerate drug diffusion to the external phase during emulsification, thereby reducing the encapsulated fraction.^89^

The polynomial equation for *Y*_2_ was as follows:

$$Y_{2}=73.73-4.78X_{1}+5.25X_{2}-2.95X_{3}-2.70X_{1}X_{3}-2.05X_{2}X_{3}+3.71{X_{1}}^{2}+1.66{X_{2}}^{2}$$

The time required to release 80% of the encapsulated drug (*T*_80_) ranged from 46.8 to 70.3 h. Increasing the polymer:drug ratio had a sustained effect on drug release, presumably due to the formation of a denser polymeric matrix that retards drug diffusion. On the other hand, a higher aqueous phase and stabilizer concentration shortened the *T*_80_. The increased stabilizer concentration likely enhanced emulsification efficiency, resulting in the formation of smaller nanoparticles with larger surface areas and thinner polymeric shells, thereby reducing diffusional path lengths and accelerating drug release.^90–92^ These trends are also attributable to lower entrapment efficiency at higher stabilizer levels, which facilitates faster drug diffusion from the nanoparticles.^93^

The polynomial equation for *T*_80_ was as follows:

$$Y_{3}=48.70-6.21X_{1}+4.19X_{2}-2.58X_{3}-4.97X_{1}X_{2}-2.20X_{1}X_{3}+5.01{X_{1}}^{2}+2.21{X_{2}}^{2}+4.49{X_{3}}^{2}$$

To validate the predictive performance of the developed models, three checkpoint batches were formulated. The observed values for particle size, entrapment efficiency, and *T*_80_ deviated by less than 5% from the predicted responses, confirming the model’s reliability (Figure 2A–C). Using numerical and graphical optimization strategies, the desired criteria (particle size <200 nm, entrapment efficiency >70%, and *T*_80_ ∼ 48 h) were employed to identify an optimal formulation. The design space was visualized via overlay plots derived from the response surface methodology (Figure 2D). The optimized formulation was defined with an aqueous phase:organic phase ratio of 3.98, a polymer:drug ratio of 8, and a stabilizer concentration of 2.5%. This formulation was selected for further physicochemical and biological evaluation.

**Figure 2:**
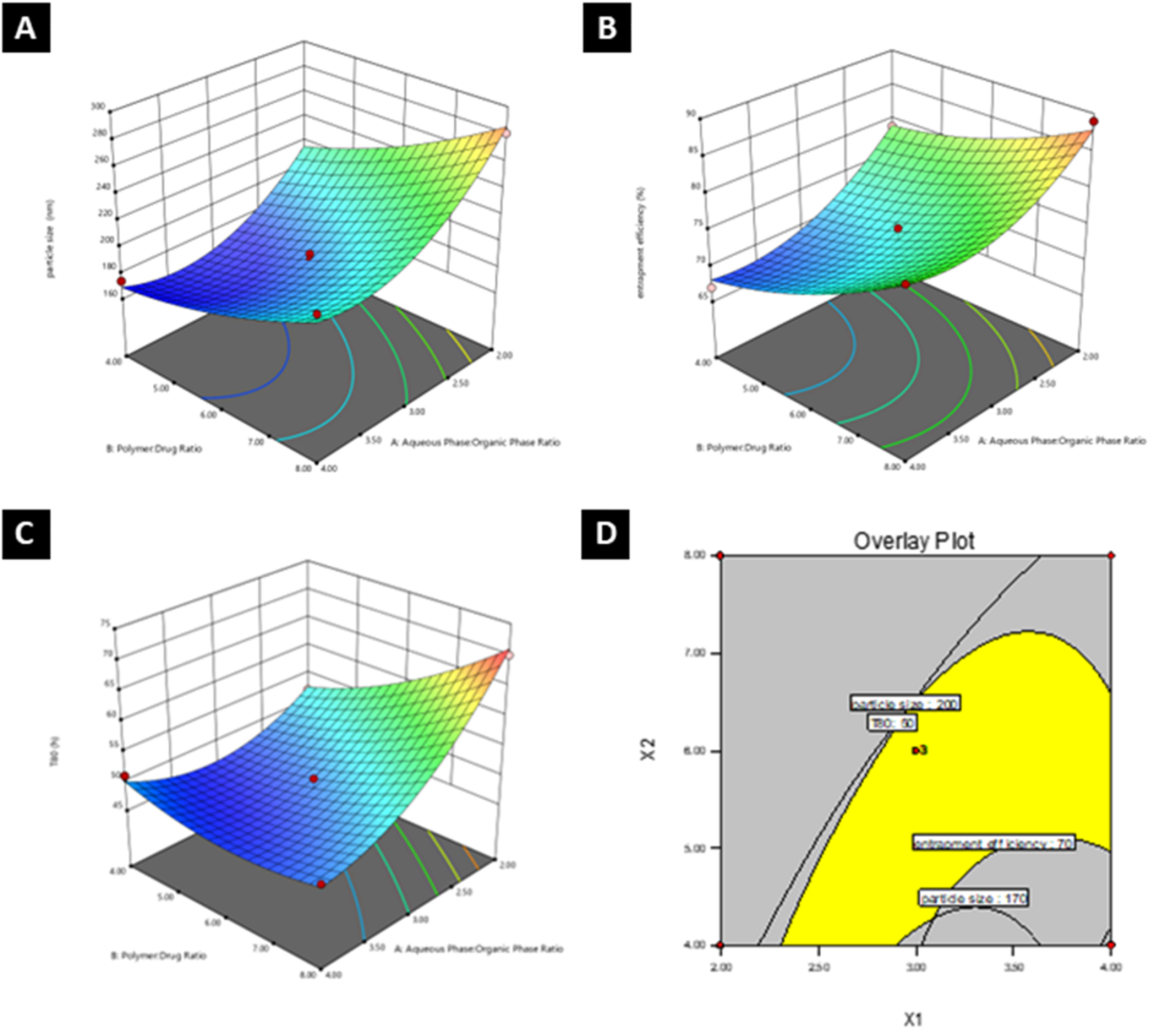
(A–C) Three-dimensional response surface plots showing the effects of formulation variables on (A) particle size (nm), (B) entrapment efficiency (%), and (C) *T*_80_ (time required to release 80% of the drug, h), generated via the Box–Behnken design. (D) Overlay plot for graphical optimization indicating that the design space satisfies the following constraints: particle size <200 nm, entrapment efficiency >70%, and *T*_80_ ∼ 48 h.

### 4.3 Physicochemical Characterization and In Vitro Drug Release

The prepared polymeric nanoparticles exhibited a small particle size and a narrow PDI, which are advantageous for enhancing drug accumulation within the tumor microenvironment via the EPR effect.^94,95^ DLS measurements revealed that the optimized 5-FU-loaded PLGA–PEG–FOL nanoparticles had a PDI of 0.119  ±  0.008 and an average particle size of 178.47  ±  3.26 nm (Figure 3A). The observed hydrodynamic size is well-suited for passive tumor targeting through the EPR effect, facilitating tumor-specific accumulation. Similar ligand-conjugated PLGA nanoparticle systems have been developed by other researchers, including Esmaeili et al., who reported particle sizes in the range of 200–210 nm.^37^ Notably, the incorporation of the folate-targeting moiety may slightly increase the particle size due to its surface orientation, but this does not compromise delivery efficiency.^96^ Zeta potential is another critical indicator of nanoparticle stability. The zeta potential of the developed formulation was measured to be −23.4  ±  0.351 mV. The high absolute value suggests a strong surface charge, which helps prevent aggregation via electrostatic repulsion. The presence of PEG contributes to steric stabilization, potentially prolonging the systemic circulation time. Moreover, a negative surface charge is generally associated with improved safety profiles in terms of cytotoxicity and genotoxicity.^97,98^

**Figure 3:**
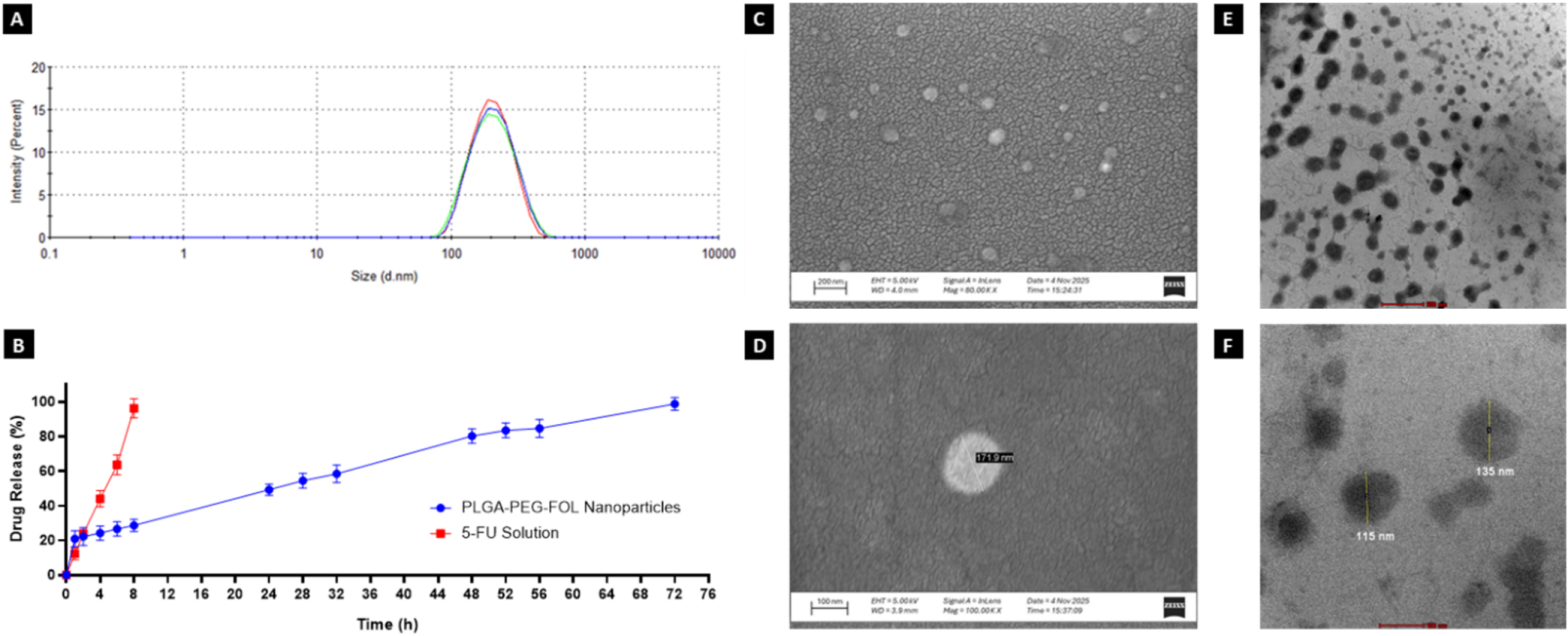
(A) Dynamic light scattering (DLS) plot of the optimized 5-FU-loaded PLGA–PEG–FOL nanoparticles, indicating a narrow particle size distribution and uniformity (*n* = 3). (B) In vitro drug release profiles of free 5-FU solution and 5-FU-loaded PLGA–PEG–FOL nanoparticles in PBS (pH 7.4) at 37 ±  0.5 °C, illustrating biphasic sustained release from the nanoparticles compared with immediate release from the free drug solution. Data are presented as the mean ± SD (*n* = 3). (C, D) SEM images of freeze-dried 5-FU-loaded PLGA–PEG–FOL nanoparticles. Panel C shows a uniform field of spherical nanoparticles at × 80,000 magnification (scale bar = 200 nm), while panel D provides a magnified view of an individual particle highlighting surface morphology (×100,000, scale bar = 100 nm). (E, F) TEM images of 5-FU-loaded PLGA–PEG–FOL nanoparticles (Tecnai 12 G2, 120 kV). Panel E shows well-dispersed spherical nanoparticles with uniform contrast (scale bar = 500 nm), whereas panel *F* presents enlarged views of individual particles with annotated diameters (scale bar = 100 nm).

Representative SEM micrographs of the optimized 5-FU-loaded PLGA–PEG–FOL nanoparticles (Figure 3C,D) showed discrete, spherical particles with smooth surfaces, confirming the successful nanoparticle formation. A textured background was observed, which is a common drying artifact arising from excipient residues (e.g., mannitol) during drop-cast sample preparation and does not reflect the nanoparticle morphology.^99,100^ ImageJ analysis of SEM images yielded an average particle diameter of 124.25 ± 17.20 nm (*n* = 15), slightly smaller than the hydrodynamic size due to the expected difference between dry-state and solution measurements.^101^ Complementary TEM images (Figure 3E,F) further confirmed the spherical morphology and nanoscale dimensions of the nanoparticles. Although some particles appeared in proximity or loosely clustered, this is a common artifact of TEM sample preparation. During grid drying, nanoparticles can migrate and concentrate due to capillary forces, surface tension-driven contraction, and partial adsorption onto the carbon-coated grid, causing them to appear closer together than in solution.^102^ Importantly, individual particles retained clear boundaries and consistent contrast, indicating that true aggregation did not occur and that the structural integrity of the optimized PLGA–PEG–FOL nanoparticles remained intact.

The DL% and EE% of the nanoparticles were calculated to be 4.89 ±  0.41% and 78.93  ±  1.05%, respectively. The in vitro release of 5-FU from the nanoparticles was evaluated via a Franz diffusion cell at 37.0  ±  0.5 °C in phosphate-buffered saline (PBS, pH 7.4). In contrast to the nanoparticle formulation, 5-FU solution (5-FU-SOL) released its entire drug content within 8 h. This rapid release can be attributed to the high solubility of 5-FU, a characteristic of BCS Class III drugs.^103^ Over time, the percentage of 5-FU released from the PLGA–PEG–FOL nanoparticles was 48.45  ±  4.263% at 24 h, 78.61 ±  5.261% at 48 h, and 98.81  ±  3.746% at 72 h. The sustained and controlled release observed from the polymeric nanoparticles, as opposed to their immediate release from the solution, suggests a uniform drug entrapment within the nanoparticle matrix. A detailed evaluation of the release profile revealed a biphasic pattern: an initial burst release of 20.96  ±  4.57% within the first hour, followed by a prolonged release phase that lasted up to 72 h (Figure 3B). The initial burst likely results from the diffusion of surface-adsorbed or weakly bound drugs, whereas the later sustained release can be attributed to gradual drug diffusion and polymer degradation via hydrolysis. This dual-phase behavior points to a release mechanism that begins with diffusion-driven desorption, followed by near-zero-order kinetics governed by polymer erosion.^104,105^

### 4.4 In Vitro Cytotoxicity Evaluation

The blank PLGA–PEG nanoparticles demonstrated excellent biocompatibility, maintaining a viability of approximately 98% across all concentrations tested. This confirmed that the polymeric carrier and PEGylation did not induce any measurable cytotoxicity in PC-3 cells, thereby validating the safety of the delivery platform itself. In contrast, treatments containing 5-FU produced a clear concentration-dependent reduction in cell viability (Figure 4A). The IC_50_ values derived from fitted dose–response curves were approximately 408 μg/mL for the 5-FU solution, 287 μg/mL for PLGA–PEG nanoparticles loaded with 5-FU, and 254 μg/mL for PLGA–PEG–FOL nanoparticles loaded with 5-FU (Figure S3). Encapsulation of 5-FU within PLGA–PEG nanoparticles substantially lowered the IC_50_ compared with the free drug, indicating enhanced intracellular delivery, most likely due to the increased stability of the encapsulated drug and improved uptake associated with nanoparticle internalization.^106^

**Figure 4:**
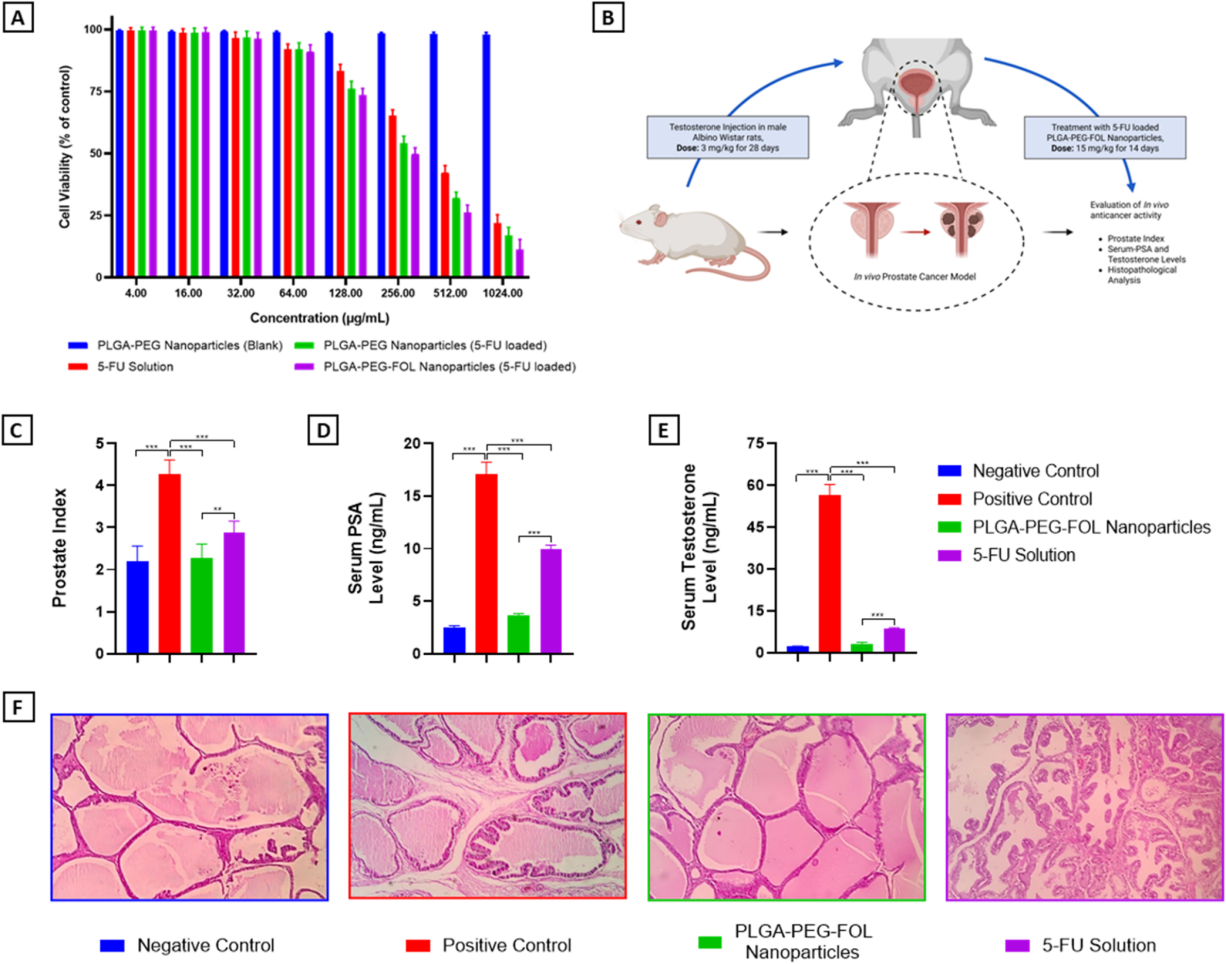
(A) Viability of PC-3 cells after 48 h of exposure to different formulations across the concentration range 2–1024 μg/mL. Blank PLGA–PEG nanoparticles maintained approximately 98% viability at all concentrations, confirming carrier biocompatibility. A concentration-dependent decrease in viability was observed for all 5-FU-containing groups, with calculated IC_50_ values of ∼408 μg/mL for 5-FU solution, ∼287 μg/mL for PLGA–PEG nanoparticles loaded with 5-FU, and ∼254 μg/mL for PLGA–PEG–FOL nanoparticles loaded with 5-FU. Data are presented as mean ± SD (*n* = 3). (B) Schematic representation of the testosterone-induced prostate cancer model in male Albino Wistar rats and the treatment protocol with 5-FU-loaded PLGA–PEG–FOL nanoparticles. (C) Prostate indices across treatment groups demonstrate the therapeutic efficacy of nanoparticle-based delivery. (D, E) Quantitative evaluation of serum prostate-specific antigen (PSA) and testosterone levels in various experimental groups. The data are presented as the means ± SDs (*n* = 8). Significant differences were determined by one-way ANOVA followed by Tukey’s posthoc test; *p* < 0.05 = *, *p* < 0.01 = **, *p* < 0.001 = ***. (F) Representative histological images (H&E staining, 40× objective; total magnification 400×) of dorsolateral prostate (DLP) tissues from the different animal groups. The sections demonstrated normal architecture in the negative control group, pathological hyperplasia in the disease control group, and varying degrees of tissue recovery in the treatment groups, highlighting the enhanced therapeutic response of the nanoparticulate formulation.

While the folate-functionalized nanoparticles exhibited a lower IC_50_ than the nontargeted PLGA–PEG-5-FU nanoparticles, the difference was relatively small. This outcome aligns with expectations for conventional monolayer cell culture systems, where every cell has direct and continuous access to the formulation. Such uniform and prolonged exposure allows even nontargeted nanoparticles to interact effectively with cell membranes through nonspecific adsorptive endocytosis, diffusion-driven contact, or fluid-phase pinocytosis. These pathways can partly overshadow the contribution of folate receptor-mediated uptake, thereby diminishing the measurable difference between targeted and nontargeted systems. In vitro culture conditions also lack several physiological barriers that exist in vivo, such as serum protein binding, vascular endothelium, and interstitial matrix density. These barriers restrict passive diffusion and nonspecific nanoparticle entry in the body, creating a scenario where receptor-mediated endocytosis becomes more influential. The observed absence of statistical significance in cytotoxicity does not necessarily indicate inefficacy of folate targeting but rather reflects the limitations of the in vitro model in replicating physiological complexity. In actual tumor microenvironments, the expression of folate receptors on cancer cell surfaces, combined with restricted nanoparticle diffusion and competitive plasma interactions, would enhance the selectivity and retention of folate-decorated nanoparticles.^107–109^ Furthermore, the ligand orientation and surface density also influence receptor binding efficiency. The relatively short PEG spacer used in this study provides steric stability but may slightly reduce the accessibility of surface-bound folate molecules under static in vitro conditions. Dynamic in vivo environments involving convective transport and cellular turnover may promote more efficient receptor–ligand interactions, leading to stronger targeting performance.^110,111^ Taken together, these findings indicate that while the cytotoxicity improvement achieved through folate conjugation appears modest under simplified in vitro conditions, the physicochemical design principles remain valid for in vivo targeting. The reduced IC_50_ trend, even without statistical significance, supports the mechanistic role of folate decoration and suggests that its benefits will likely become more apparent in the presence of biological barriers that hinder passive uptake.

Two critical conclusions can be drawn. First, the nanoparticle platform itself is highly biocompatible and does not compromise cell viability at the concentrations tested, confirming its safety for further biological evaluation. Second, the incorporation of 5-FU into PLGA–PEG nanoparticles significantly enhances cytotoxicity relative to the free drug, while folate conjugation offers an additional but subtle improvement that is expected to gain clinical importance in physiologically relevant environments where receptor-targeted mechanisms dominate. Overall, these results highlight both the promise and the limitations of in vitro cytotoxicity assays, emphasizing the necessity of corroborating such findings with in vivo studies to determine the true therapeutic impact of folate-mediated targeting in prostate cancer treatment.

### 4.5 In Vivo Anticancer Evaluation

Figure 4B provides a schematic overview of the in vivo experimental design for establishing a testosterone-induced prostate cancer model in male Albino Wistar rats and subsequent therapeutic intervention with 5-FU-loaded PLGA–PEG–FOL nanoparticles. Prostate carcinogenesis was induced through the subcutaneous administration of testosterone at a dosage of 3 mg/kg/day for 28 consecutive days. Following confirmation of prostate enlargement, the animals were stratified into treatment groups. The therapeutic group was orally administered 5-FU-loaded PLGA–PEG–FOL nanoparticles at a dosage of 15 mg/kg/day for 14 days. Figure 4C illustrates the impact of 5-FU solution and 5-FU-loaded PLGA–PEG–FOL nanoparticles on the prostate index in testosterone-induced prostate cancer in rats. There were no significant differences in body weight across the groups, indicating that the treatments did not adversely affect general animal health (Figure S4). Compared with the negative control group, the testosterone-treated positive control group presented a significant increase (nearly 2-fold) in the prostate index, confirming prostate enlargement and successful model induction. Compared with the positive control, treatment with 5-FU-loaded PLGA–PEG–FOL nanoparticles significantly (p ≤ 0.05) reduced the prostate index. Specifically, the nanoparticle formulation resulted in approximately 46.73% inhibition, whereas the free 5-FU solution resulted in 32.72% inhibition. This differential effect suggests that the nanoparticulate system offers superior therapeutic efficacy, likely due to sustained drug release and enhanced tumor accumulation via folate receptor–mediated targeting.^112^ Interestingly, the prostate indices of the rats treated with the nanoparticle formulation did not significantly differ from those of the negative control group, whereas a significant difference persisted in the 5-FU solution–treated group. These observations reinforce the superior therapeutic potential of the targeted nanoparticle formulation and align with findings from previous studies.^113^

The enhanced therapeutic efficacy of the folate-functionalized nanoparticles is widely accepted to arise from folate receptor-mediated endocytosis, a well-established and extensively studied mechanism in targeted drug delivery. FRs are abundantly expressed on prostate cancer cells, allowing high-affinity binding and selective internalization of folate-decorated nanoparticles. Once bound, these nanoparticles undergo clathrin-independent endocytosis and are trafficked into endosomal compartments, where controlled polymer degradation and endosomal escape enable gradual intracellular release of 5-FU. This receptor-mediated uptake significantly enhances intracellular drug accumulation, circumvents efflux-mediated resistance, and improves cytotoxic potency compared with passive diffusion of the free drug. Concurrently, PEGylation confers steric stabilization, minimizes opsonization, and prolongs systemic circulation, promoting tumor accumulation via the EPR effect. Together, these passive and active targeting mechanisms synergistically contribute to the superior in vivo efficacy observed with the PLGA–PEG–FOL nanoparticles.

Serum biomarker levels, specifically PSA and testosterone, were measured to further assess the therapeutic efficacy. As shown in Figure 4D,E, both biomarkers were significantly elevated in the positive control group following testosterone administration compared with those in the negative control group. After 14 days of treatment, the PSA level was reduced by approximately 78.83% in the nanoparticle-treated group and by 41.67% in the free 5-FU-treated group. This substantial reduction in PSA, a key clinical indicator of prostate cancer progression, highlights the enhanced efficacy of the targeted nanoparticle system. A similar trend was observed for serum testosterone levels. Compared with the positive control, treatment with 5-FU-loaded PLGA–PEG–FOL nanoparticles resulted in a 94% reduction, whereas the free drug achieved an 85% reduction. At the end of the study, no significant difference in PSA or testosterone levels was detected between the nanoparticle-treated and negative control groups, whereas significant differences remained for the free 5-FU group. These results confirm the improved therapeutic performance of the nanoparticle system in regulating prostate cancer-related biomarkers.

Histological examination of prostate tissues was performed via H&E staining to evaluate treatment-induced morphological changes (Figure 4F). In the negative control group, the dorsolateral prostate displayed a well-organized tubular structure lined with a single layer of cuboidal epithelial cells and characteristic luminal secretions.^114,115^ In contrast, the positive control group exhibited extensive hyperplasia and dysplasia with prominent adenomas and adenocarcinomas, increased tubular diameter, multilayered epithelium, and papillary projections. These alterations closely mimic the histopathological features of human prostatic dysplasia.^116,117^ Treatment with 5-FU-loaded PLGA–PEG–FOL nanoparticles substantially reversed these pathological changes. A marked reduction in hyperplastic and dysplastic lesions was observed with the restoration of near-normal architecture. In comparison, the free 5-FU–treated group showed only modest improvements and abnormal tissue structures remained evident. These findings support the conclusion that the nanoparticle formulation not only enhances drug delivery but also results in superior histological correction, likely due to improved cellular uptake and sustained therapeutic release.^118–120^

While these outcomes provide strong proof of concept for targeted chemotherapy using PLGA–PEG–FOL nanoparticles, it is important to recognize the scope and inherent limitations of the experimental design. Although the testosterone-induced prostate cancer model used in this study provides a reliable and physiologically relevant system to mimic androgen-driven prostatic hyperplasia, it represents an early stage, short-term model rather than a fully developed neoplastic state. The 28-day induction and 14-day treatment phases were selected to allow controlled assessment of therapeutic efficacy and biomarker modulation within a reproducible time frame. However, this design does not capture long-term tumor progression, metastatic dissemination, or recurrence patterns. Future studies will therefore aim to extend treatment duration and employ more advanced orthotopic or xenograft models to better evaluate long-term pharmacodynamic behavior and translational performance of the targeted nanoparticle system.

## 5 Conclusion

This study presents a comprehensive approach to the development of a targeted nanoparticle-based drug delivery system for prostate cancer therapy. A folic-acid-functionalized amphiphilic copolymer, PLGA–PEG–FOL, was synthesized via carbodiimide chemistry and confirmed via FT-IR spectroscopy, which indicated successful amide bond formation between the functional groups of PLGA, PEG, and folic acid. The PLGA–PEG–FOL copolymer was subsequently used to formulate 5-FU-loaded nanoparticles via a modified emulsification–solvent evaporation technique.

Formulation optimization was performed via a Box–Behnken experimental design to evaluate the effects of formulation variables, including the aqueous-to-organic phase ratio, polymer-to-drug ratio, and surfactant concentration, on key response parameters. The optimized formulation exhibited a particle size of 178.47 ± 3.26 nm, a polydispersity index of 0.119 ± 0.008, a zeta potential of −23.4 ± 0.35 mV, an entrapment efficiency of 78.93 ± 1.05%, and a drug loading of 4.89 ± 0.41%. The sustained release profile of the nanoparticles followed a biphasic pattern characterized by an initial burst release attributed to surface-associated drugs, followed by a prolonged release phase governed by diffusion and polymer degradation kinetics.

The in vitro cytotoxicity study conducted on PC-3 prostate cancer cells demonstrated enhanced cellular uptake and cytotoxic potential of the folate-targeted nanoparticles, as evidenced by a 1.6-fold reduction in the IC_50_ compared with that of free 5-FU. These findings suggest efficient folate receptor-mediated endocytosis and improved intracellular drug accumulation. In vivo evaluation using a testosterone-induced prostate cancer model in male Albino Wistar rats further validated the therapeutic potential of the formulation. Compared with the disease control and free 5-FU groups, the 5-FU-loaded PLGA–PEG–FOL nanoparticle groups presented significantly lower prostate indices, serum PSA levels, and serum testosterone levels. Histopathological assessment confirmed the reversal of hyperplasia and dysplasia in prostate tissues, which is consistent with effective anticancer activity.

These results collectively demonstrate that folate-conjugated PLGA–PEG nanoparticles can serve as a potent and targeted delivery platform for 5-FU, improving therapeutic efficacy and potentially reducing systemic side effects. The nanosystem offers favorable physicochemical stability, controlled drug release, enhanced tumor selectivity, and in vivo performance. Future work should explore detailed pharmacokinetic and biodistribution studies, immunogenicity profiling, and scale-up potential to facilitate translation into preclinical and clinical applications in prostate cancer management.

## Supplementary Material



## Notes

The Supporting Information contains additional experimental data that complement the findings presented in the main manuscript. It includes The Supporting Information is available free of charge at https://pubs.acs.org/doi/10.1021/acsomega.5c07466.

Differential scanning calorimetry (DSC) thermograms and X-ray diffraction (XRD) profiles comparing pure 5-FU with 5-FU-loaded PLGA–PEG–FOL nanoparticles, confirming the transition of the drug from crystalline to amorphous form; Supplementary cytotoxicity concentration–response curves for PC-3 cells are provided to support the in vitro evaluation; and Body-weight monitoring data for all treatment groups are also included to assess systemic tolerability (PDF)
