# Supplementary material for: Folate-Functionalized Polymeric Nanoparticles for 5‑Fluorouracil Delivery to Prostate Cancer: Physicochemical and In Vitro/In Vivo Characterization
Source: ACS Omega. 2025 Dec 25;11(1):810–25. doi: 10.1021/acsomega.5c07466 (PMC12809314; doi:10.1021/acsomega.5c07466)
Supplement: Supplementary file 1 [file ao5c07466_si_001.pdf]

**Folate-functionalized Polymeric Nanoparticles for 5-fluorouracil Delivery to Prostate Cancer: Physicochemical and In Vitro/In Vivo Characterization**

Bhumi Bhatt [a], Gajanan Kalyankar [a], Bhavin Vyas [a], Manisha Lalan [b],

Nimeet Desai [c], Lalitkumar K. Vora [d, *], Pranav Shah [a, *]

[a] Maliba Pharmacy College, Uka Tarsadia University, Gopal Vidyanagar, Bardoli-Mahuva Road, Tarsadi, Gujarat, 394350, India

[b] Parul Institute of Pharmacy and Research, Faculty of Pharmacy, Parul University, P.O. Limda, Waghodia, Vadodara, Gujarat, 391760, India

[c] Department of Eye and Vision Science, Institute of Life Course and Medical Sciences, University of Liverpool, 6 West Derby Street, Liverpool L7 8TX, United Kingdom

[d] School of Pharmacy, Queen's University Belfast, 97 Lisburn Road, Belfast BT9 7BL, United Kingdom

***** *Corresponding Authors***

PS: pranav.shah@utu.ac.in

LKV: L.vora@qub.ac.uk

**Supplementary Data**

## 1.1 Differential scanning calorimetry

Differential scanning calorimetry (DSC) was employed to evaluate the thermal behaviour and physical state of the optimized 5-FU-loaded PLGA-PEG-FOL nanoparticles in comparison to pure 5-FU. DSC thermograms were recorded using a DSC-60 thermal analyser (Shimadzu Corporation, Japan). The instrument was calibrated for temperature and heat flow using high-purity indium and zinc standards. Accurately weighed samples were sealed in aluminium pans and heated from 25 °C to 300 °C at a constant rate of 10 °C/min under a nitrogen purge flow of 20 mL/min. The resulting thermal profiles were analysed to assess phase transitions, crystallinity, and potential drug–polymer interactions [1,2].

**Result:** To investigate the physical state of the encapsulated drug and potential drug–polymer interactions, DSC analysis was performed. **Figures S1** present the DSC thermograms of free 5-FU and 5-FU-loaded PLGA-PEG-FOL nanoparticles, respectively. Pure 5-FU exhibited a sharp endothermic peak at 283.49 °C, characteristic of its crystalline nature [3,4]. In contrast, this distinctive melting peak was absent in the thermogram of the drug-loaded nanoparticles, suggesting that 5-FU was no longer present in a crystalline state. The disappearance of the endothermic peak implies that the drug was molecularly dispersed within the polymeric matrix in an amorphous form, potentially contributing to improved solubility and controlled release [5].

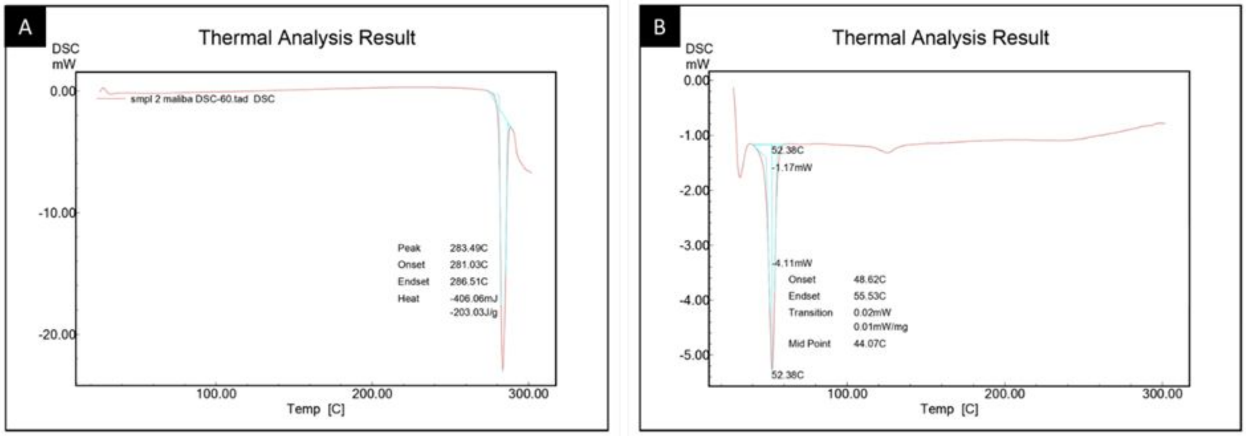


**Figure S1.** DSC thermograms of (A) pure 5-FU and (B) 5-FU-loaded PLGA-PEG-FOL nanoparticles. The thermogram of pure 5-FU exhibits a sharp endothermic peak at 283.49°C corresponding to its melting point, confirming its crystalline nature. In contrast, the thermogram of the nanoparticle formulation shows the absence of a distinct melting peak, indicating the transformation of 5-FU into an amorphous state upon encapsulation within the polymeric matrix.

## 1.2 X-ray diffraction analysis

X-ray diffraction analysis was conducted to investigate the crystalline characteristics of pure 5-FU and the optimized 5-FU-loaded PLGA-PEG-FOL nanoparticles. Diffractograms were recorded at room temperature using a MiniFlex XpC diffractometer (Rigaku, Japan) equipped with a Cu Kα radiation source (λ = 1.5406 Å), operating at 40 kV and 40 mA with a nickel filter. Samples were mounted on zero-background sample holders and scanned continuously over a 2θ range of 5° to 40°, with a step size of 0.01° and a scan speed of 1 s per step. The resulting diffraction patterns were analysed using DIFFRAC.EVA software (version 8, Bruker) to evaluate crystallinity and possible changes in the solid-state properties of the drug upon encapsulation [6].

**Result:** XRD analysis further corroborated the DSC findings. As shown in **Figure S2**, the diffractogram of pure 5-FU displayed multiple sharp peaks, confirming its crystalline structure. Upon incorporation into the PLGA-PEG-FOL nanoparticle system, these intense diffraction peaks were markedly reduced or absent, indicating a loss of crystallinity. The reduced peak intensity in the drug-loaded nanoparticles supports the conclusion that 5-FU exists predominantly in an amorphous or molecularly dispersed state within the nanocarrier system [7,8]. Together, these findings confirm the successful encapsulation of 5-FU and the transition of the drug from crystalline to amorphous form, a transformation that may contribute to enhanced drug solubility, stability, and sustained release behaviour.

A

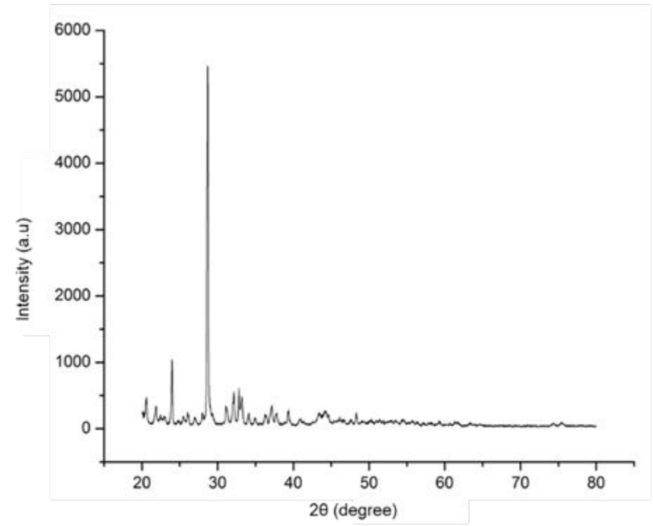


B

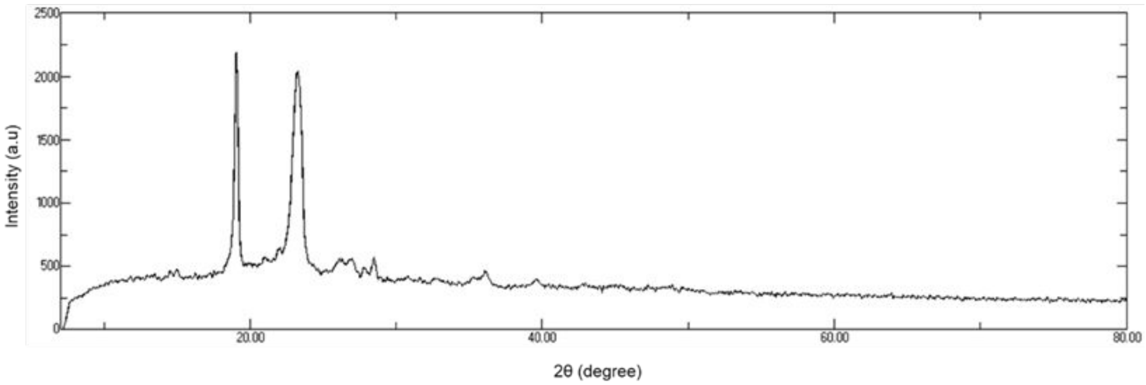

**Figure S2.** XRD patterns of (A) pure 5-FU and (B) 5-FU-loaded PLGA-PEG-FOL nanoparticles. The diffractogram of pure 5-FU exhibits sharp and intense peaks, particularly in the 2θ range of 20° to 35°, indicating its crystalline structure. In contrast, the XRD profile of the nanoparticle formulation shows a substantial reduction in peak intensity and sharpness, suggesting a transition of 5-FU into an amorphous or molecularly dispersed state within the PLGA-PEG-FOL matrix.

### 1.3 Dose–Response and $IC_{50}$ Determination

PC-3 cells were seeded in 96-well plates and treated with increasing concentrations of 5-FU solution, PLGA-PEG nanoparticles, or PLGA-PEG-FOL nanoparticles for 48 h. Cell viability was assessed using the MTT assay, and absorbance was measured at 570 nm using a microplate reader. Viability (%) was calculated relative to untreated controls. Dose–response curve (**Figure S3**) was generated by fitting the data to a nonlinear sigmoidal model (four-parameter logistic regression). The $IC_{50}$ value for each formulation was obtained from the fitted curve as the concentration producing 50% cell viability.

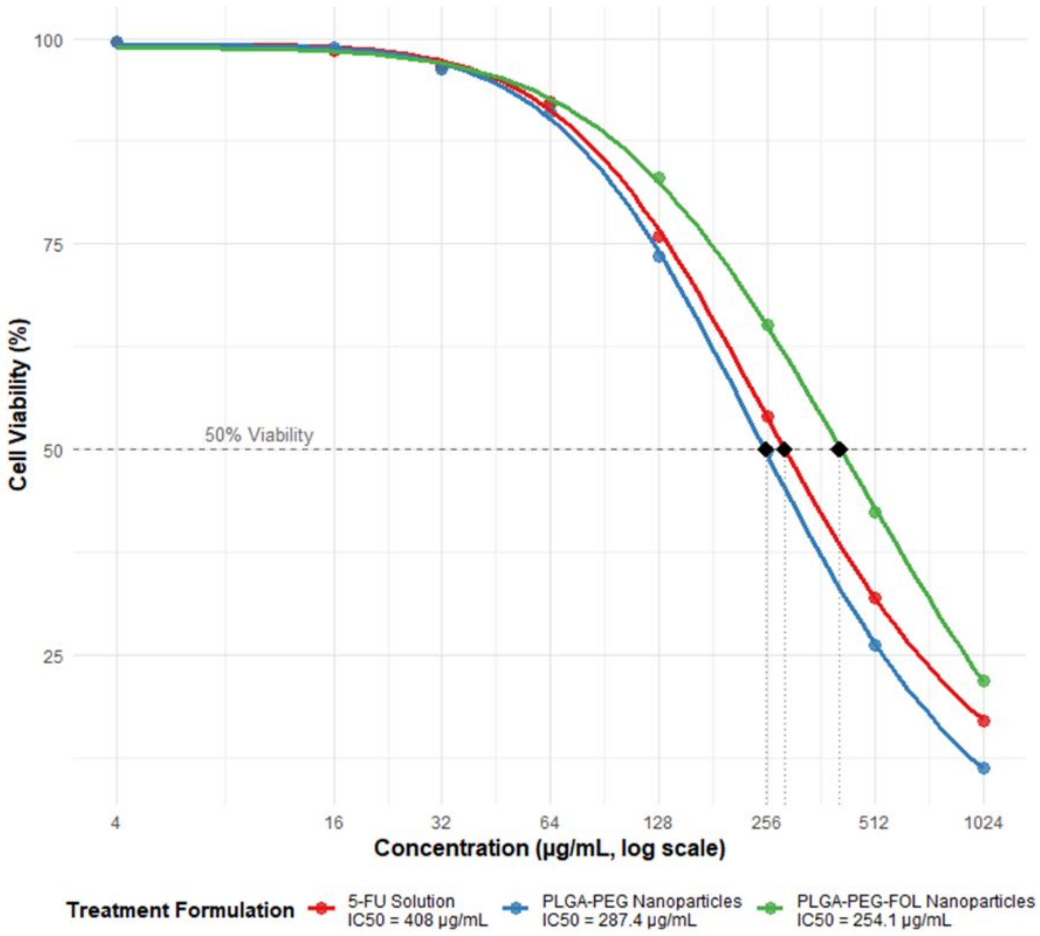


**Figure S3.** Concentration–response curves for PC-3 cells treated with 5-FU solution, PLGA-PEG nanoparticles loaded with 5-FU, and PLGA-PEG-FOL nanoparticles loaded with 5-FU.

### 1.4 Body Weight Monitoring During the Treatment Period

To assess overall health status and potential systemic toxicity, the body weights of all experimental animals were monitored throughout the induction and treatment phases. The mean ± SD values for each group (n = 8) are presented in **Figure S4**. Statistical analysis revealed a significant reduction in body weight only between the disease control and negative control groups (**** $p < 0.0001$). No statistically significant differences were observed for the 5-FU solution or PLGA-PEG-FOL nanoparticle-treated groups, confirming that both treatments were well tolerated and did not induce systemic toxicity.

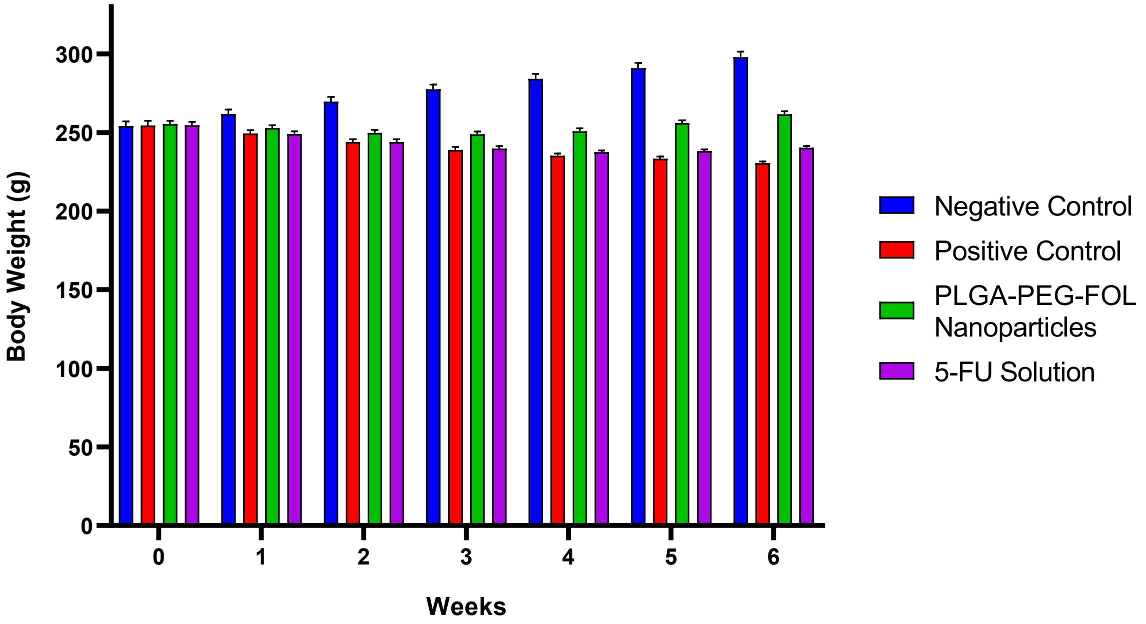


**Figure S4.** Changes in the mean ± SD body weight of Wistar rats during the induction and treatment phases across experimental groups: (A) Negative Control, (B) Disease Control, (C) 5-FU Solution, and (D) 5-FU-Loaded PLGA-PEG-FOL Nanoparticles (n = 8 per group). A significant decrease in body weight was observed only between the positive-control and negative-control groups (**** $p < 0.0001$), whereas no significant differences were detected for the 5-FU solution or PLGA-PEG-FOL nanoparticle groups.

### Supplementary References


[1] H. Li, Y. Tong, L. Bai, L. Ye, L. Zhong, X. Duan, Y. Zhu, Lactoferrin functionalized PEG-PLGA nanoparticles of shikonin for brain targeting therapy of glioma, Int J Biol Macromol 107 (2018) 204–211. https://doi.org/10.1016/j.ijbiomac.2017.08.155.

[2] P.J. Shah, M.P. Patel, J. Shah, A.B. Nair, S. Kotta, B. Vyas, Amalgamation of solid dispersion and melt adsorption techniques for augmentation of oral bioavailability of novel anticoagulant rivaroxaban, Drug Deliv Transl Res 12 (2022) 3029–3046. https://doi.org/10.1007/s13346-022-01168-9.

[3] E.A.K. Nivethaa, S. Dhanavel, V. Narayanan, C.A. Vasu, A. Stephen, An in vitro cytotoxicity study of 5-fluorouracil encapsulated chitosan/gold nanocomposites towards MCF-7 cells, RSC Adv 5 (2015) 1024–1032. https://doi.org/10.1039/C4RA11615A.

[4] F. Jubeen, A. Liaqat, M. Sultan, S. Zafar Iqbal, I. Sajid, F. Sher, Green synthesis and biological evaluation of novel 5-fluorouracil derivatives as potent anticancer agents, Saudi Pharmaceutical Journal 27 (2019) 1164–1173. https://doi.org/10.1016/j.jsps.2019.09.013.

[5] N. Kamaly, B. Yameen, J. Wu, O.C. Farokhzad, Degradable Controlled-Release Polymers and Polymeric Nanoparticles: Mechanisms of Controlling Drug Release, Chem Rev 116 (2016) 2602–2663. https://doi.org/10.1021/acs.chemrev.5b00346.

[6] M. Yadav, J. Sarolia, B. Vyas, M. Lalan, S. Mangrulkar, P. Shah, Amalgamation of Solid Dispersion and Melt Adsorption Technique: Improved In Vitro and In Vivo Performance of Ticagrelor Tablets, AAPS PharmSciTech 22 (2021) 257. https://doi.org/10.1208/s12249-021-02138-z.

[7] E. Berbel Manaia, M. Paiva Abuçafy, B.G. Chiari-Andréo, B. Lallo Silva, J.A. Oshiro-Júnior, L. Chiavacci, Physicochemical characterization of drug nanocarriers, Int J Nanomedicine Volume 12 (2017) 4991–5011. https://doi.org/10.2147/IJN.S133832.

[8] A. Kumar Sahdev, C.J. Raorane, M.A. Ali, K. Mashay Al-Anazi, R.K. Manoharan, V. Raj, A. Singh, Chitosan-Folic Acid-Coated Quercetin-Loaded PLGA Nanoparticles for Hepatic Carcinoma Treatment, Polymers (Basel) 17 (2025) 955. https://doi.org/10.3390/polym17070955.
